# Ultrafast evolution of electric fields from high-intensity laser-matter interactions

**DOI:** 10.1038/s41598-018-21711-4

**Published:** 2018-02-19

**Authors:** R. Pompili, M. P. Anania, F. Bisesto, M. Botton, E. Chiadroni, A. Cianchi, A. Curcio, M. Ferrario, M. Galletti, Z. Henis, M. Petrarca, E. Schleifer, A. Zigler

**Affiliations:** 1 Laboratori Nazionali di Frascati, 00044 Frascati, Italy; 2 Racah Institute of Physics, Hebrew University, 91904 Jerusalem, Israel; 3 University of Rome Tor Vergata and INFN, 00133 Rome, Italy; 4 Istituto Superior Tecnico de Lisboa, 1049-001 Lisbon, Portugal; 5 University of Rome Sapienza, 00185 Rome, Italy

## Abstract

The interaction of high-power ultra-short lasers with materials offers fascinating wealth of transient phenomena which are in the core of novel scientific research. Deciphering its evolution is a complicated task that strongly depends on the details of the early phase of the interaction, which acts as complex initial conditions. The entire process, moreover, is difficult to probe since it develops close to target on the sub-picosecond timescale and ends after some picoseconds. Here we present experimental results related to the fields and charges generated by the interaction of an ultra-short high-intensity laser with metallic targets. The temporal evolution of the interaction is probed with a novel femtosecond resolution diagnostics that enables the differentiation of the contribution by the high-energy forerunner electrons and the radiated electromagnetic pulses generated by the currents of the remaining charges on the target surface. Our results provide a snapshot of huge pulses, up to 0.6 teravolt per meter, emitted with multi-megaelectronvolt electron bunches with sub-picosecond duration and are able to explore the processes involved in laser-matter interactions at the femtosecond timescale.

## Introduction

Multi-Terawatt laser pulses with femtosecond duration have opened new horizons in research of nonlinear transient phenomena like astrophysics in laboratory^1,2^, particle acceleration^3–5^, material science^6,7^, surface phenomena^8^ (breakdown and surface manipulation), nuclear^9^ and medical physics^10,11^. More specifically, the transfer of energy from the laser field to the particles in the bulk of the target lies in the heart of all the processes and acts as a complex initial condition. It is therefore fundamental to have a clear and precise understanding of the interaction process in the transient regime, where all the customary models that assume thermal equilibrium are stretched to their proper end of justification and beyond.

When a laser pulse operating at relativistic intensities (${I}_{L}\gtrsim {10}^{18}$ W/cm^2^) irradiates a solid target, a force driven by the laser field is produced and accelerates electrons up to relativistic velocities^12–16^. These hot electrons propagate through target and then are ejected^17^. Some of them (fast electrons) are energetic enough to completely escape the target charging it rapidly. The unbalanced positive charge left on target leads thus to the formation of a strong electric potential that locks the majority of hot electrons close to the target^16^. The hot electrons bounce back and forth and continue to ionize the matter creating a plasma. Such a recirculation generates huge electromagnetic pulses^18–20^, up to some teravolt per meter (TV/m) depending on the laser intensity, whose spectral content is constituted by two main components. The first one consists in ultra-short EM pulses in the THz domain^21,22^ and is mostly related to the current associated with fast electrons. Their propagation, measured through coil-like structures^23^ or wires^24,25^, occurs on tens-picosecond time scale. The second component is due to the neutralization currents that flows through into the target to balance the net positive charge left on it. The duration is on the nanosecond timescale and corresponds to well-known pulses in the GHz domain whose features have been widely investigated^26–28^.

A complete picture of the several processes involved in laser-target interactions is experimentally complicated to obtain since different diagnostic techniques are usually needed. The release of the fast electrons accompanied by the emission of ultra-short EM pulses, in particular, has not yet been experimentally provided. The major obstacle, in this case, is obtaining detailed and reliable data that closely follows the fields and changes in the transient interaction process that evolves at the sub-picosecond scale and up to several picoseconds^16^. So far experimental evidences were obtained with high-resolution measurements of the plasma density near the target surface^29^, by probing the plasma quasi-static magnetic fields^30,31^ or the emitted electric pulses^22,32^. The probing can be alternatively conducted by tracing down the escaping particles, e.g. protons^33,34^ and electrons^35,36^.

Here we report, for the first time, single-shot snapshots with femtosecond resolution of the ultra-short EM pulses and fast electrons emitted during the interaction between a multi-terawatt laser and a sharp metallic target. Our detection system adopts a non-destructive and single-shot temporal diagnostics based on Electro-Optical Sampling^37^ (EOS) able to directly follow the evolution of the emitted electromagnetic pulses and charged particles flow generated during and after the interaction. The results, corroborated by Particle-In-Cell (PIC) simulations, provide a more complete picture of interaction processes at the sub-picosecond timescale and highlight the ultra-fast dynamics involved during and soon after the transfer of energy from the laser pulse to the target.

## Results

### Experimental setup

**Figure 1:**
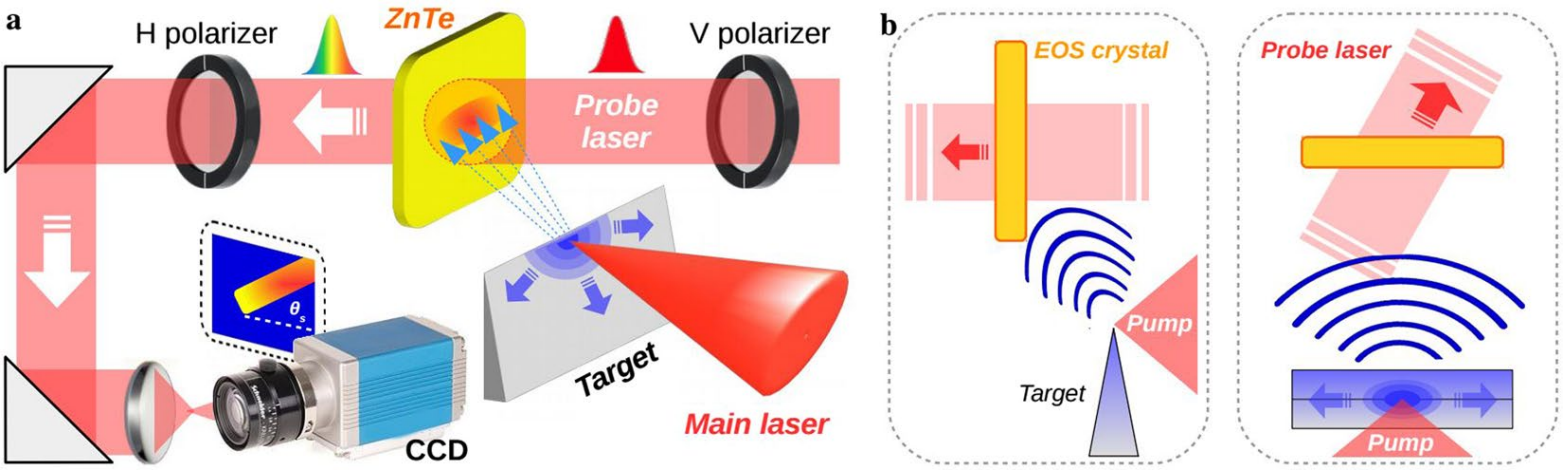
Setup of the experiment. (**a**) The FLAME laser is focused on a sharp metallic target producing electrons that escape from it. The unbalanced charge (blue region) gradually spreads on target and generates a radiation pulse that induces a local birefringence on the electro-optic crystal. A linearly polarized probe laser simultaneously crosses the crystal and its transverse profile is locally modulated along the red rectangle shape rotated by *θ*_*s*_. (**b**) Side view (left) and top view (right) of the EOS setup. The ZnTe crystal is located 1 mm downstream and above the metallic target where the FLAME laser is focused. The emitted radiation (blue wave) propagates in free space and is directly detected by the EOS diagnostics.

The experiment is performed with FLAME, the 100 TW laser system available at the SPARC_LAB test-facility^38^. The setup is depicted in Fig. 1(a). The laser, with intensity *I*_*L*_ ≈ 10^18^ W cm^−2^, is focused on a stainless steel sharp target (0.7 *μm*-thick commercial razor blade). The temporal diagnostics is based on the EOS, i.e. on the electro-optic effect induced into a nonlinear crystal (that becomes birefringent) by an externally applied electric field. Such a birefringence is able to modulate the polarization of an incoming laser pulse impinging onto the crystal proportionally to the electric field amplitude. Our system employs a 10 × 10 mm^2^ ZnTe electro-optic crystal (500 *μm*-thick) and a 35 fs probe laser derived from the main system^39,40^. We show in Fig. 1(b) a detailed view of the EOS setup. The probe laser enters into the ZnTe crystal with *θ*_*i*_ = 28° incidence angle and realizes a spatial encoding of the temporal profile of the EM pulse along the probe transverse profile^41^. An optical delay-line line is used to change the delay of the probe with respect to the main laser in order to monitor the interaction at different times. The probe laser is finally detected by a CCD camera. The EOS resolution is determined by several factors like the the probe laser duration and the electro-optic crystal (type and thickness) itself^42^. For our setup we expect a sub-100 fs temporal resolution thus we operate on the same timescale of the interaction process, determined by the duration of the driving laser pulse.

**Figure 2:**
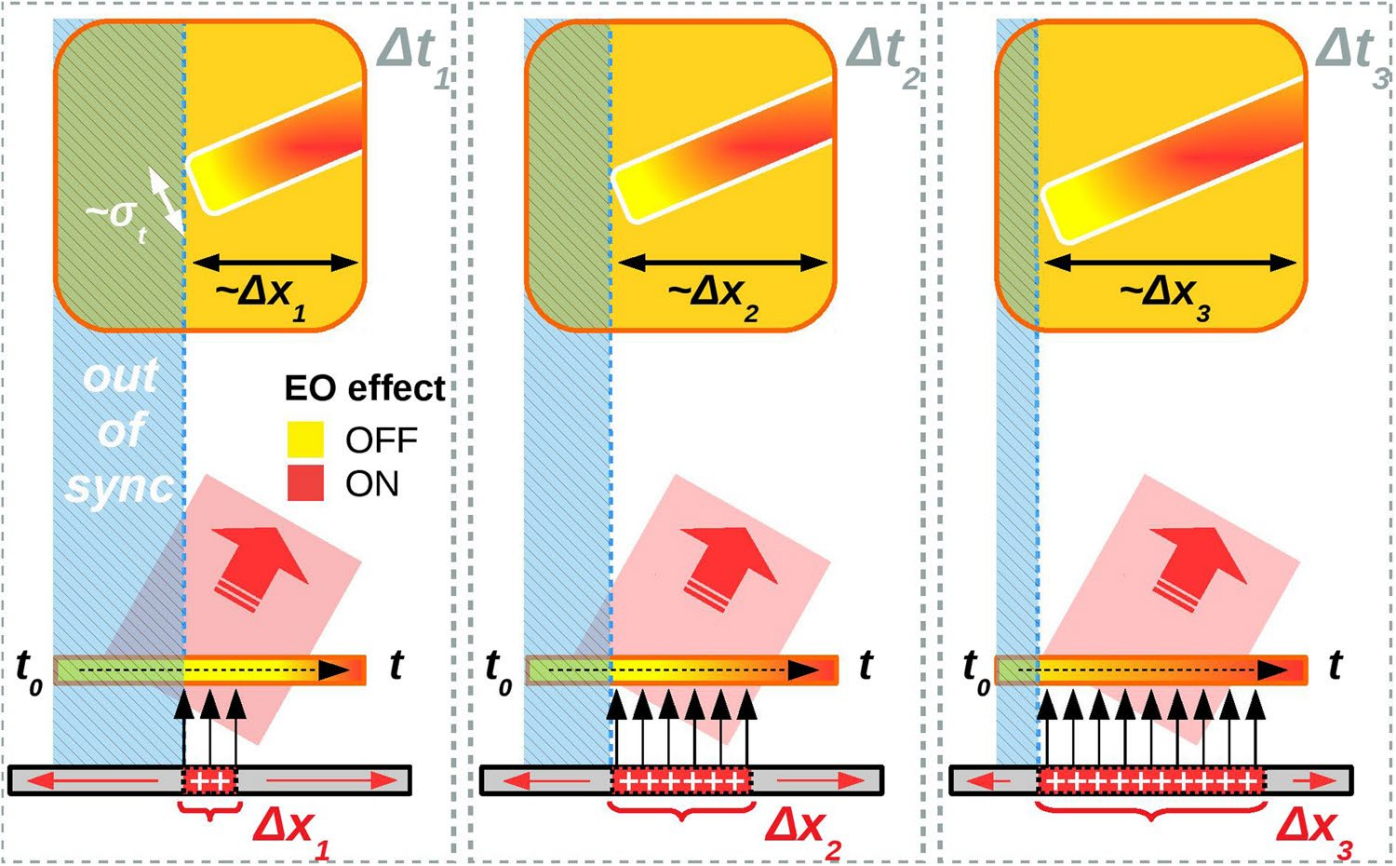
Electro-optic encoding and EM pulse detection. Signal detection at different delays (Δ*t*_1_ < Δ*t*_2_ < Δ*t*_3_) between the probe and the main pulses. The probe laser enters into the crystal from left to right (earlier times on the left). The blue shaded area represents the size of the crystal not affected by EO effect (probe in advance with respect to the EM pulse). The probe is temporally synchronized with the EM pulse (with duration *σ*_*t*_) only on the remaining part, i.e. only a part of its front-wave (whose size is proportional to the target emitting area Δ*x*) is detected.

Figure 2 shows the electro-optic encoding realizing along the laser probe transverse profile. The EM pulse, once emitted, propagates toward the EOS crystal located at 1 mm distance. Here it is imprinted on the probe transverse profile along a tilted cigar-like shape (red rectangle) whose thickness is directly proportional to its duration (denoted by *σ*_*t*_). Indicating with *v*_*L*_ ≈ *c*/sin*θ*_*i*_ (*v*_*T*_ ≈ *c*) the velocity at which the laser (EM pulse) spans over the entire crystal and *c* the vacuum speed of light, the angle of the electro-optic signal with respect to the *x*-axis is *θ*_*s*_ = arctan(*v*_*T*_/*v*_*L*_) ≈ 25°.

### Electro-optic encoding

The features of the resulting electro-optic signals are mainly due to the size of EM emitting source and the geometry involved in the experiment. The probe laser enters into the crystal with an angle and spans it horizontally from left to right. The earlier time (*t*_0_) corresponds to the left side of the crystal. From Fig. 2 an external electric field impinging on the crystal in advance with respect to the probe laser is not encoded and thus no signal is produced (blue shaded area). It follows that, depending on the delay Δ*t* between the main and probe pulses, only a part of the EM pulse wave-front is detected (the rightmost one) and the resulting signal is not symmetric. The physical size of the emitting source (red area with width Δ*x*) determines finally the horizontal extent of the signals. Wider is the source, wider is the area of the crystal that becomes birefringent due to the electro-optic effect.

As reported in Methods, the amount of induced birefringence strongly depends on the orientation of the external electric field with respect to the crystallographic axes. In our specific case, it is maximized when the EM pulse points normally to the crystal surface (black arrows). As depicted in Fig. 2, it is possible to probe how the effective size (Δ*x*) of the target emitting area changes with time. We expect, in particular, that for wider sources the electro-optic signal appears elongated toward left since the temporal synchronization with the probe realizes also at earlier times. From the horizontal extent of the detected signal (detected at different delays Δ*t*) the velocity at which the emitting area grows with time can be thus inferred.

### Detection of ultra-short EM pulses

**Figure 3:**
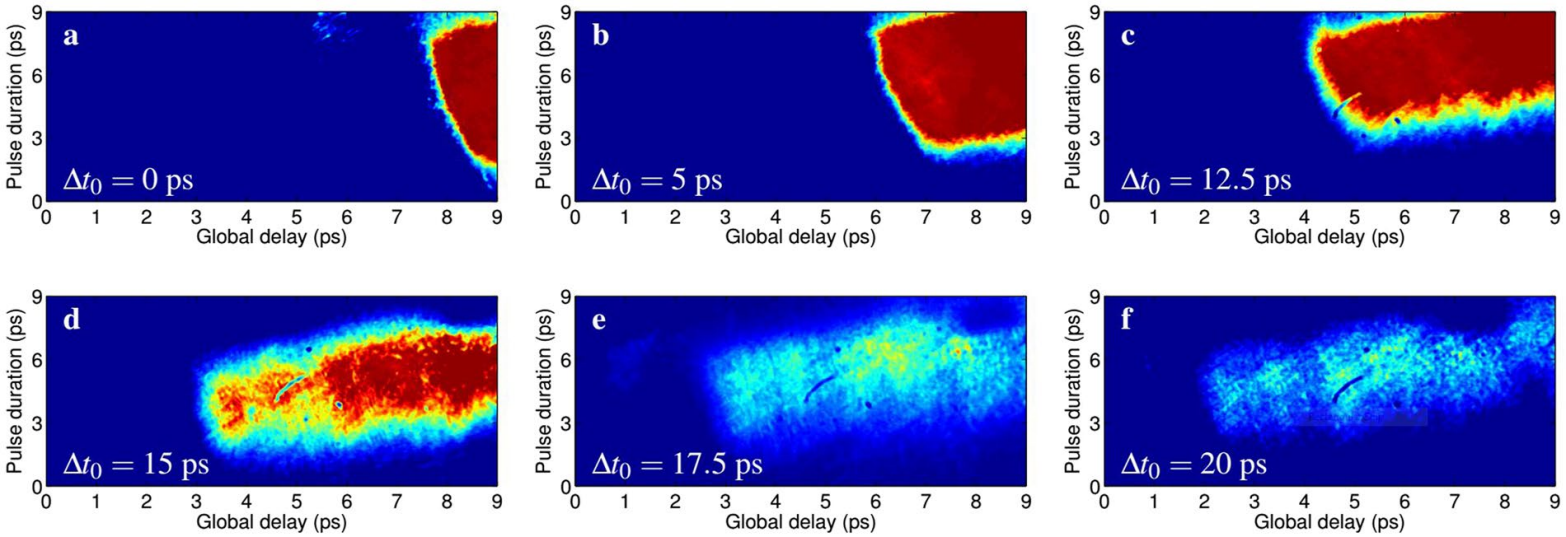
Timeline of the radiation pulse evolution. Experimental signals obtained by focusing the main laser onto a wedged target at different probe laser delays (Δ*t*_0_). The *x*-axis represents the relative probe arrival time (*t*) associated to each pixel. The absolute delay between the main pulse and the *i*-th pixel is thus Δ*t*_0_ + *t*_*i*_.

Figure 3 shows a series of single-shot electro-optic signals obtained by focusing the FLAME laser on the sharp tip of the metallic blade. The use of blades in place of common planar foils allows the emitted pulses to freely propagate in space toward the EOS (where they are actually detected) and to not block the probe laser before entering into the crystal, placed very close to the interaction point. In each snapshot the probe laser is progressively delayed by steps of 2.5 ps to investigate the evolution of the pulses emitted by the target at late times. As one can see, by delaying (anticipating) it with respect to the FLAME laser, the signal gradually shifts toward left (right) and decreases (increases) in amplitude. The *x*-axis (global delay) represents the probe laser arrival time on crystal. By indicating with Δ*t*_0_ the delay between the main and probe pulses and with *t*_*i*_ the relative time associated to the *i*-th pixel (the probe laser enters into the crystal from left to right, thus *t* = 0 is located at the leftmost side), the absolute delay between the main pulse and the pixel itself is exactly Δ*t*_0_ + *t*_*i*_. The EM pulse duration, instead, is retrieved from the vertical thickness of the electro-optic signal (see Fig. 2). One can see that in all cases the signal shape resembles the one depicted in Fig. 1(a) with *θ*_*s*_ ≈ 24°, close to what expected.

**Figure 4:**
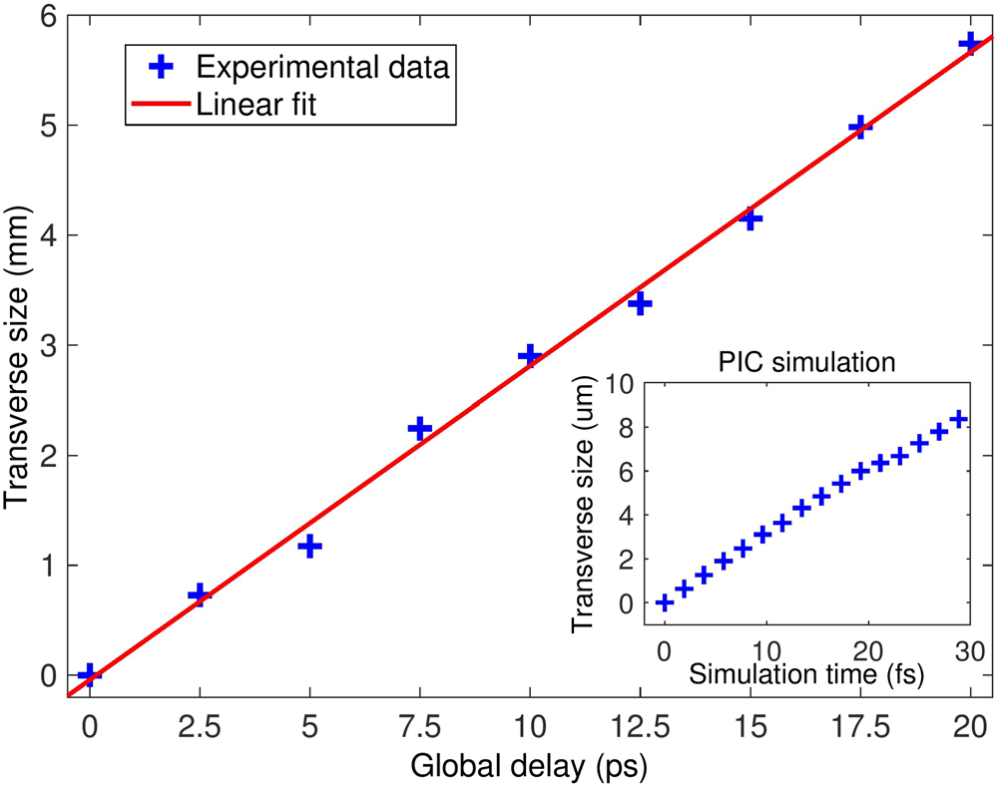
EM pulse transverse size evolution Radial size (+) of the detected ultra-short EM pulses as a function of the probe laser delays reported in Fig. 3. The values reported on the *y*-axis are calculated with respect to radial size of the signal shown in Fig. 3(a), took as reference. A linear fit indicates that the radial size increases at speed (0.94 ± 0.03)*c*. (inset) Radial size (°) evaluated with PIC simulations.

The timeline reported by Fig. 3 highlights the dynamics of the target charging process. After the release of the first electrons, an unbalanced positive charge is left on it. At the beginning the charge is mainly concentrated in a tiny area (approximately corresponding to the laser spot size^16^) and results in a large surface charge density (Fig. 3(a)). The target actually acts like an antenna emitting electromagnetic pulses. With time the entire target charges^20,27^. Sectors far from the interaction point, indeed, become positively charged since their electrons are attracted and flow toward the interaction point. As a consequence the overall surface charge density lowers and the weaker pulses emitted by these sectors produce smaller electro-optic signals (Fig. 3(f)). By measuring the horizontal extent (in terms of pixels, whose physical size is known) of the detected signals we can also retrieve the charge spreading velocity or, similarly, the velocity at which the antenna emitting area grows with time. Indeed, since a larger emitting area produces wider EM radiation, its radial extent is directly proportional to the horizontal size of the detected signal. Figure 4 shows the relative increase (with respect to Fig. 3(a), took as reference) in the radial size of the signals reported in Fig. 3 as a function of the delay between the probe and main laser pulses. From the experimental data we calculate a charge spreading velocity of (0.94 ± 0.03)*c*, in agreement with other experiments^23,24,43^. The inset shows a comparison, obtained through PIC simulations (described in the following), of the fast spread of the EM fields on the target surface. The resulting speed is (0.95 ± 0.03)*c*.

As a result of the combined emission by all the charged sectors of target, the typical signature depicted in Fig. 1(a) is obtained. Its thickness is directly proportional to the average duration of the detected radiation that, for the signals reported in Fig. 3, is 6 ±1 ps. As a final consideration, the same snapshots highlight that there is a detectable signal even after 20 ps. Such a statement does not mean that the pulse duration is 20 ps but is rather a consequence of the retarded emission of further pulses (with same duration) by the outer sectors of target. The picture, instead, indicates that the process is mainly dictated by the currents coming from the outer regions of target and lasts in approximately 20 ps. After that time the resulting signals are too low to be detected by the EOS.

### Role of laser energy deposition on target

The interaction of intense short laser pulses with solid targets results in a strong heating of the interacting particles. As a consequence some high-energy electrons are ejected while others spread and dissipate their energy inside of it. The entire process is directly correlated to the intensity of the interacting laser pulse and can be described in terms of the average temperature of the ejected hot electrons^27,44^ that, in our experimental conditions, is expected to be *T*_*h*_ ≈ 500 keV.

**Figure 5:**
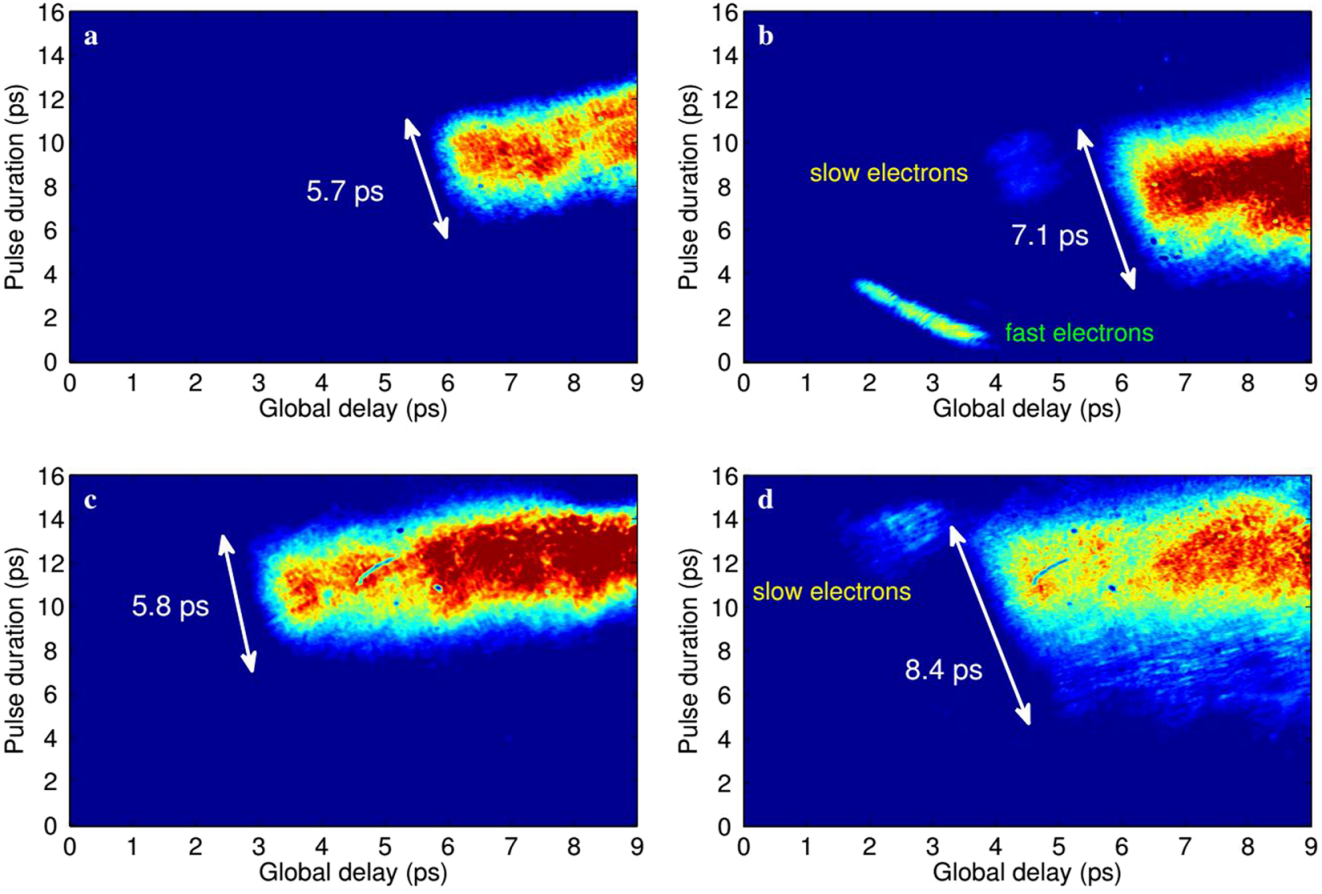
Electro-optic signals for different experimental conditions. (**a**,**b**) Single-shot snapshots obtained when the laser is focused on the tip (0.0.7*μm* thickness) of the target with 50% (**a**) and 100% (**b**) energy. The signature of two escaping electron bunches is also detected. (**c**,**d**) The laser pulse with a fixed energy (50%) is now focused on the tip (**c**) and bulk (6 *μm* thickness, d) of the target, resulting in partial and full energy deposition, respectively. The probe laser was delayed by 4 ps in (**c**,**d**) with respect to (**a**,**b**), resulting in a signal shift toward left.

To investigate the generation and evolution of the emitted radiation pulses we performed further measurements analyzing the effects related to the amount of laser energy deposited on the target itself. So far it has been suggested that the both amplitude and duration of the emitted EM radiation should increase by increasing laser pulse energy^20,27^. We examined this conjecture by setting-up the FLAME laser energy *E*_*L*_ at 50% (1 J) and 100% (2 J) energy. We show the experimental results in Fig. 5(a) and (b), respectively. In the first case we measured a pulse duration of 5.7 ps. As expected, when the energy is doubled both the amplitude and duration of the EM pulse increase. The duration of the pulse in Fig. 5(b), in particular, increased up to 7.1 ps (larger vertical size of the signal). The escaping electrons are clearly visible in the same snapshot, highlighting two distinct components at different energies and charges. These quantities will be properly quantified in the following.

A similar behavior on the pulse duration is expected when the laser is focused, in turn, on the tip or on the bulk (about 20 *μm* far from the tip, roughly corresponding to about 6 *μm* of material thickness) of the metallic target. Figure 5(c) and (d) point out this aspect. In both snapshots the laser energy was set to 50% (≈1 J) and the probe laser was delayed by 4 ps, resulting in a signal shift toward left. With such a configuration, Fig. 5(c) is actually a delayed replica of Fig. 5(a) and the resulting pulse duration is approximately the same (5.8 ps). Similarly, Fig. 5(d) now resembles Fig. 5(b) since a larger deposition of energy into the target leads again to the development of an electromagnetic field with longer duration (8.4 ps). For this delay only the low-energy component of the escaping electrons is detected, while the most energetic one is already out of the EOS time window. These results are pointing out that the dynamics involved in laser-target interactions is strongly dependent on the amount of laser energy that is deposited on target.

### Release of fast electrons

So far the release of accelerated electrons whose energy spectrum consists of two distinct components, as the one observed in Fig. 5(b), was studied with theoretical models^45,46^ and numerical simulations^16^. Recently we have experimentally measured the quantity and temporal evolution of the escaping electrons and their dependence on the target shape^39^. While several competing processes are involved in the acceleration of the fastest one, the lower energy part is mainly controlled by plasma expansion and charge recycling occurring on the target. The experimental result obtained in Fig. 5(b) represents the first measurement ever done which shows both the emitted radiation pulse and the forerunner escaping electrons, providing a complete picture of all processes involved.

Due to the encoding mechanism and to the employed geometry of the experimental setup, the escaping electrons and the emitted EM pulses produce different signatures. As we reported in previous works^39,40^, the electro-optic traces associated to particles moving at relativistic speeds present characteristic narrow tilted shapes. At lower energies the electro-optic signals become broader due to the larger opening angle of the associated Coulomb field^37^ (∝ 1/*γ*, with *γ* the relativistic Lorentz factor). We have estimated the average electron energy by using the EOS as a time of flight (TOF) monitor^41^. The amount of charge extracted charge, instead, is directly correlated to the signal amplitude (see Methods). In Fig. 5(b) approximately 2 nC of high-energy electrons are located on the left and concentrated in a narrow tilted structure whose duration (determined by the width of the signal) is of the order of 400 fs (fwhm). Their average energy is 7 MeV. The low energy component of escaped electrons, being slower, reaches the crystal at a later time (about 2 ps) and shows a broader structure containing about 0.3 nC charge. For such a electronic population we have calculated, based on its overall TOF, a mean energy of about 0.6 MeV, slightly above the expected temperature *T*_*h*_ of hot electrons that are locked close to the target surface.

An order of magnitude estimate of the amount of electrons that escape from target can be calculated with a simple model by taking into account all the electrostatic forces involved in the interaction^46^. Being *N*_*total*_ = *ηE*_*L*_/*k*_*B*_*T*_*h*_ the total number of hot electrons accelerated, with *η* (typically $\gtrapprox \mathrm{10 \% }$) the fraction of laser energy absorbed by hot electrons and *k*_*B*_ the Boltzmann constant, the fraction *x* of the *N*_*total*_ laser-accelerated electrons can be evaluated by equating the minimum electron energy required to escape from the electrostatic potential that develop onto the target surface. In formulas

1 $$\frac{{\rm{I}}{\rm{n}}\,x}{x}=-\frac{{r}_{e}}{{r}_{L}}\frac{{m}_{e}{c}^{2}}{{k}_{B}{T}_{h}}{N}_{total},$$

where *r*_*e*_ is the classical electron radius, *r*_*L*_ ≈ 12 *μm* the laser spot size and *m*_*e*_ the electron rest mass. In our case we expect *N*_*total*_ ≈ 2.5 × 10^12^ and a *x* ≈ 0.8% laser conversion efficiency in escaping electrons, i.e. approximately 3 nC charge. If compared to the overall charge we have experimentally measured, these numbers result in good agreement.

### Temporal duration and amplitude scaling laws

**Figure 6:**
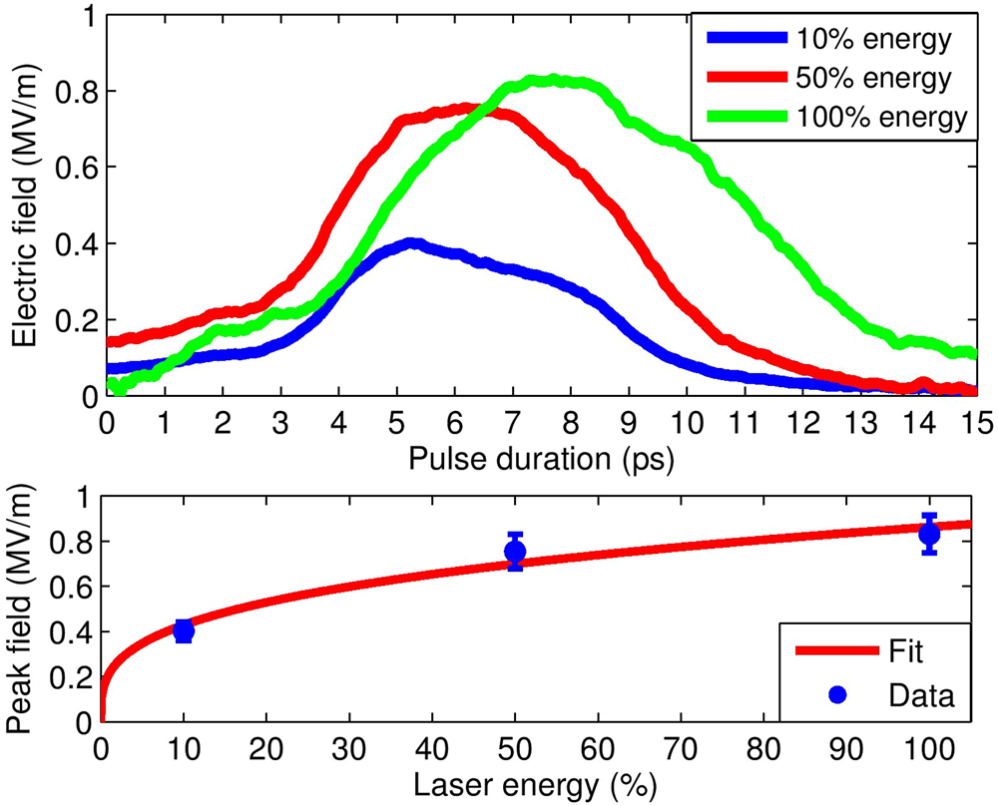
Detected EM pulses. (Top) The red (green) profile of the radiation pulse *E*_*T*_ is retrieved from the vertical signal thickness in Fig. 5(a) at 50% laser energy (Fig. 5(b), 100% energy). The (fwhm) duration is 5.7 ps (7.1 ps). When the laser energy is lowered down to 10% the duration decreases to about 5 ps (blue). (Bottom) Scaling of *E*_*T*_ peak amplitudes with laser energy. The fit is calculated according to the power law *y* = *a* ⋅ *x*^*b*^, with *b* = 0.30 ± 0.06.

The magnitude of the radiation pulse impinging onto the EOS crystal is retrieved by quantifying the amount of birefringence induced into the ZnTe crystal and sampled by the probe laser (more details in Methods). As previously pointed out, its duration is retrieved by measuring the vertical extension of the electro-optic signal. In Fig. 6 we compare the resulting fields generated when 10% (blue), 50% (red) and 100% (green) of the FLAME laser energy is focused on target. The last two cases refer to the snapshots reported in Fig. 5(a) and (b), respectively. We see that as the energy increases, there is a moderate rise of the field amplitude while its lifetime grows from about 5 ps to 5.7 ps and then up to 7.1 ps (fwhm). Figure 6 also shows the scaling of the peak electric field *E*_*T*_ (blue points) as a function of the laser energy on target *E*_*L*_. By fitting with a power law ${E}_{T}=a\cdot {E}_{L}^{b}$ we find *b* = 0.30 ± 0.06, indicating that any further increase in the laser energy only produces a moderate enhancement of the radiated field.

It is instructive to compare the radiated electromagnetic pulses with the quantity of fast electrons extracted from target. For this purpose a simple estimate can be obtained according to an analytical model based on a radially confined surface charge set up by laser accelerated electrons^47^. Being *Q*_*e*_ ≈ 2 nC the charge of the fast electron bunch measured in Fig. 5(b), the positive surface charge *Q*_*e*_ induced on the target surface leads to a surface charge density ${\sigma }_{T}={Q}_{e}/\pi {r}_{L}^{2}$ and, in turn, an electric field *E*_*T*_ = *σ*_*T*_/*ε*_0_ ≈ 0.6 TV/m. Such a field allows to escape only few electrons, i.e. exactly the ones with energies $E\gtrsim {Q}_{b}\mathrm{/2}\pi {\varepsilon }_{0}{r}_{L}\approx 3$ MeV. As described in the following, the amplitude of the resulting field on target well matches the one resulting from PIC simulations and the one detected with the EOS in Fig. 6 (green line), if the attenuation of the field with distance (∝ *r*^−2^) is considered.

## Discussion

**Figure 7:**
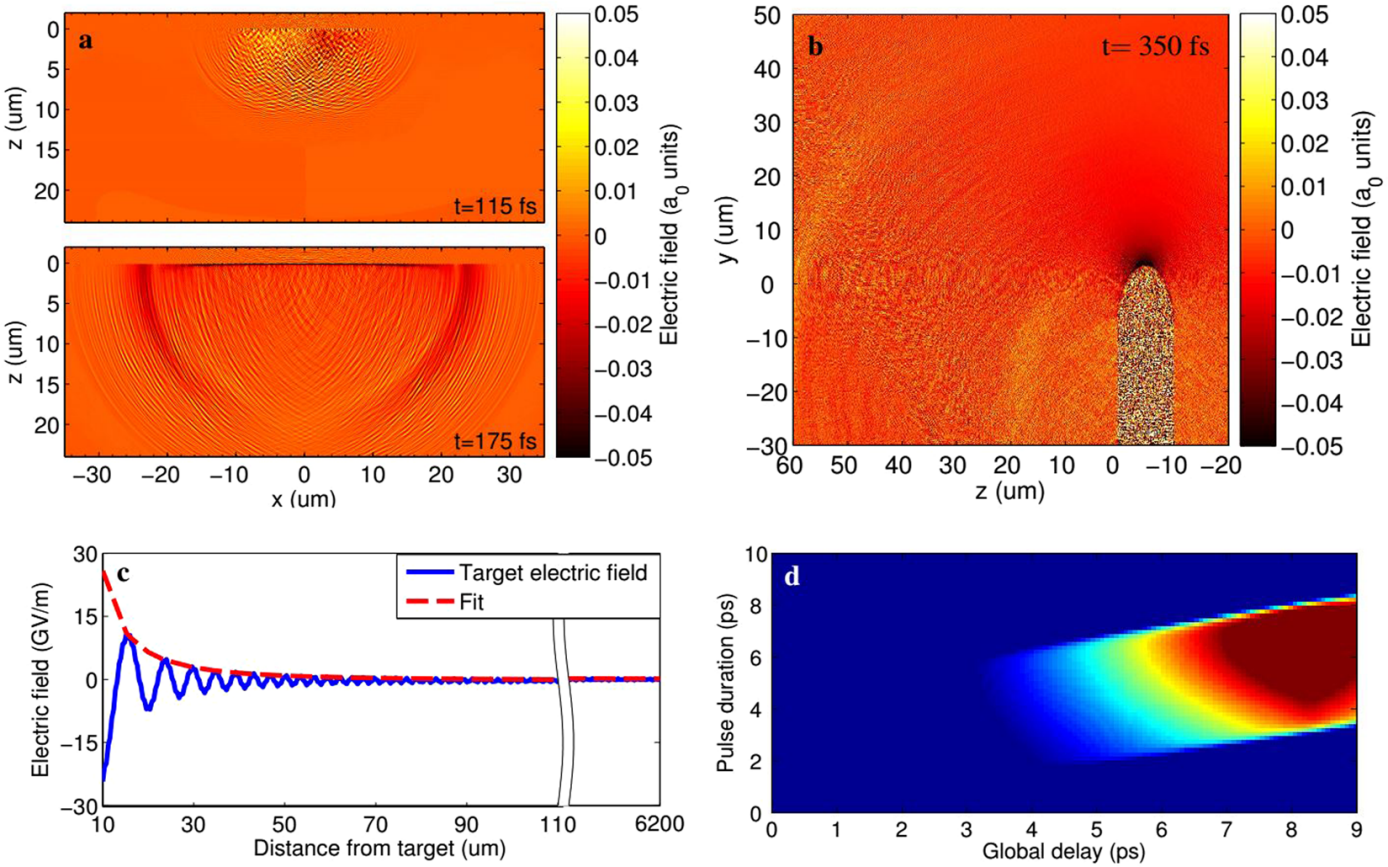
Particle-In-Cell simulations. (**a**) Electric field *E*_*T*_ radiated 115 fs and 175 fs after the interaction with the laser. The intensity is plotted in terms of the laser strength parameter *a*_0_. The transverse size of the field emitter grows as the unbalanced charge spreads on target. (**b**) Side view for the field emitted at 350 fs. The geometry of the simulated wedged target is also shown. (**c**) Extrapolated field. The fit at distance *z* is calculated according to the power law *E*_*T*_(*z*) = *a* ⋅ *z*^*b*^, with *b* = − 1.9 ± 0.2. (**d**) Simulated EOS snapshot as detected by the CCD. The radiation in (a,b) is propagated toward and through the EOS crystal, where it is sampled by the probe laser.

For a time-resolved analysis of the interaction process we used the 2D PIC code TURBOWAVE^48^. The target surface conditions were simulated self-consistently making use of measured temporal and transverse profiles of the laser pulse. Figure 7(a) shows two frames simulating the radiated field 115 fs and 175 fs after the laser-target interaction. The electric field is plotted in units of the laser strength parameter *a*_0_. The increasing of the charged emitting surface is clearly visible, with its radius growing approximately at speed of light as anticipated in Fig. 4. A maximum field strength of 0.12 *a*_0_ ≈ 0.72 TV/m is reached close to the target. As highlighted in Fig. 7(b), obtained 350 fs after the laser interaction, the radiated field is mainly emitted with an angle toward left, i.e. toward the EOS crystal.

We calculated in Fig. 7(c) the strength of the radiated field in correspondence of the electro-optic crystal (where it is measured) and we found that it drops with distance as ≈ *r*^−1.9^. Such scaling allows to extrapolate the magnitude of the electric field close to the target surface by considering that the distance between the target itself and the point of the crystal where the signal is actually emitted is *r* = 6.2 ± 0.1 mm (see Methods). It means that the 0.8 MV/m peak field obtained in Fig. 6 (100% laser energy, green line) translates in 0.60 ± 0.02 TV/m on target, close to what predicted by PIC simulations. In order to show how such electric field is actually detected by the EOS diagnostics, we report in Fig. 7(d) a simulation of the detected EOS signal. It is obtained by using, as input, the radiation as calculated by the PIC simulation that is then propagated toward and through the EOS crystal where it is sampled by the probe laser. The simulated snapshot reproduces a similar output to what experimentally measured in Fig. 5(c).

In conclusion we provided femtosecond resolution measurements that allow to operate on the same time scale of the process, determined by the duration of the driving laser pulse. In our experiment we observed the temporal evolution of the electromagnetic pulses emitted from metallic targets after the interaction with an high-intensity short pulse laser. Their lifetime of the order of several picoseconds with amplitudes of the order of 0.8 MV/m in correspondence of the EOS crystal. From this value we have inferred a field strength of approximately 0.6 TV/m on target, in agreement with PIC simulations. The provided snapshots also detected the forerunner fast particles that escaped from target and provide a more complete physical picture of the entire interaction process. Our study opens the way to perform many new time-resolved experiments with the goal to have a closer and more complete vision of the phenomena involved in laser-matter interactions.

## Methods

### Laser system and experimental area

FLAME is a 100 TW Ti:Sapphire chirped-pulse amplification (CPA) laser system providing 35 fs (fwhm) duration pulses with 60 nm bandwidth centered at *λ*_*L*_ = 800 nm with energies up to 3 J on target at 10 Hz repetition rate. The laser beam, after compression and temporal optimization by means of two acoustic-optic modulators (Fastlite Mazzler and Dazzler), is focused by an *f*/10 off-axis parabolic mirror with *f* = 1 m focal length. A Shack-Hartmann wavefront sensor and a deformable mirror are used in order to remove aberrations coming from the CPA chain and keep most of the energy (≈60%) in the main lobe of the focal spot (12 *μm* fwhm). The resulting contrast ratio (intensity in the main pulse divided by the intensity of the pre-pulse) is of the order of 10^9^ by considering the pedestal some nanoseconds before the main pulse. The laser transport line from the compressor to the interaction chamber is realized in a high vacuum environment (10^−6^ mbar) in order to avoid self-focusing effects and reduce contaminations that could affect the experiment. The probe laser used for the EOS diagnostics is provided by splitting a small fraction (50 mJ) of the main beam before the last multi-pass amplifier. It follows a specific transport line and is re-compressed by a dedicated compressor, providing 35 fs pulses. The synchronization of the main and probe lasers in correspondence of the EOS crystal is obtained by means of an *α*-cut BBO crystal installed on the ZnTe holder. The time overlapping is then retrieved by using a 3 fs resolution delay-line and looking for light emission by second-harmonic generation (SHG).

### Electro-Optic effect in Zinc Telluride

The ZnTe crystal has a zincblende cubic structure and is optically isotropic if no external electric field is applied, i.e. it has only one refractive index *n*_0_ (≈2.85 for wavelength *λ*_*L*_ = 800 nm). When an external electric field ***E***(*t*) is applied, its impermeability tensor modifies as ${\eta }_{ij}=$ ${\eta }_{ij}^{0}+{\sum }_{k}{r}_{ijk}E{(t)}_{k}$, where *r*_*ijk*_ is the tensor that describes the linear electro-optic effect and *E*_*k*_ the *k*-component of the electric field ***E***(*t*). The tensor *η* is symmetric, hence *r*_*ijk*_ = *r*_*jik*_ and it is convenient to replace the first two indices *i*,*j* by a single index^49^. A ZnTe crystal cut along the (110)-plane has moreover an high degree of symmetry and thus only one independent electro-optic coefficient *r*_41_ = *r*_52_ = *r*_63_ ≈ 4.2 × 10^−12^ m/V.

**Figure 8:**
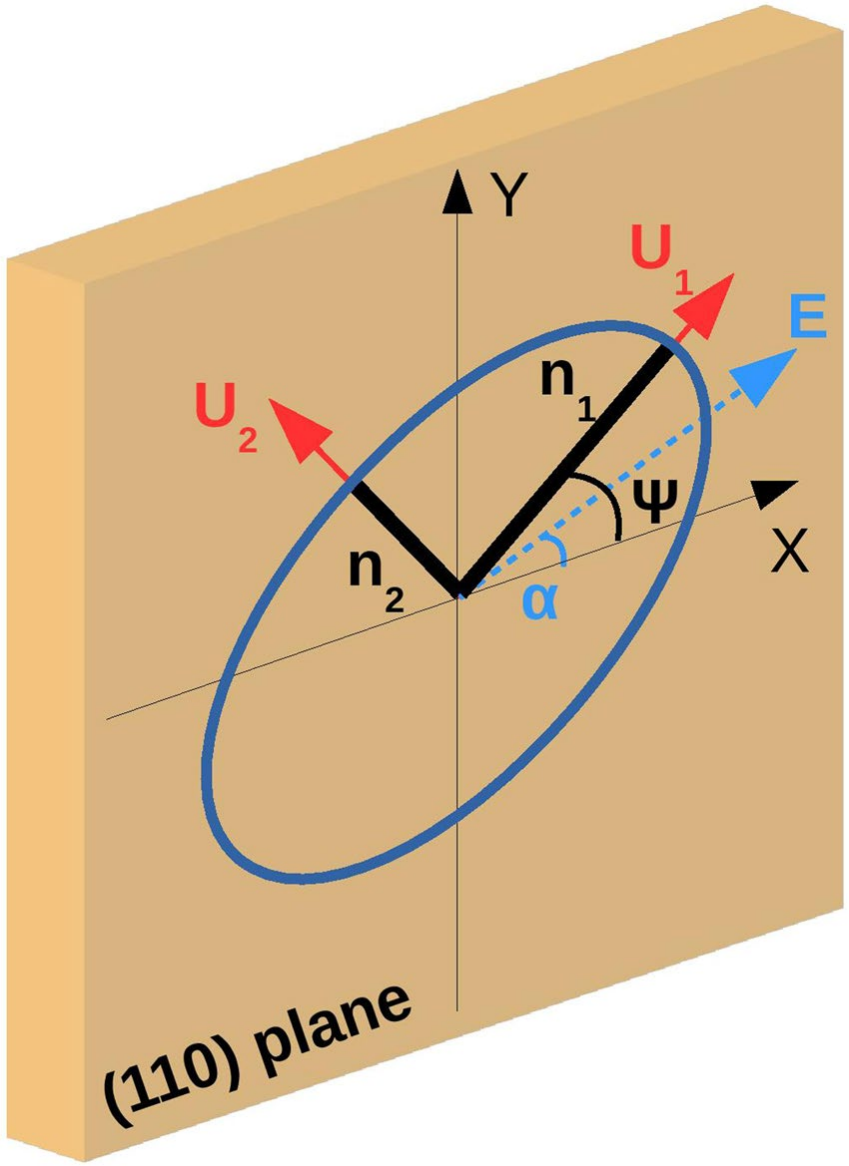
ZnTe refractive index ellipsoid. The (110) plane in the ZnTe crystal and its coordinate system (**X, Y**). If no external electric field is applied the crystal is isotropic with refractive index *n*_0_. An electric field **E** (blue arrow), with angle *α* with respect to the crystallographic **X** axis, induces the electro-optic effect. The refractive index ellipsoid projected onto the (110)-plane is sketched as an ellipse rotated by Ψ(*α*) whose main axes, parallel to (**U**_**1**_, **U**_**2**_), represent the new refractive indices *n*_1_ and *n*_2_ of the crystal.

Figure 8 shows that these indices are related to the two induced principal axes **U**_1,2_ (perpendicular to each other). Being *α* the angle between the externally applied electric field and the crystallographic **X** axis, the resulting ellipse (whose semi-axes are **U**_1,2_) is rotated by Ψ(*α*). As a result the crystal becomes birefringent, i.e. characterized by two different refractive indices. Considering that ${r}_{41}E\ll \mathrm{1/}{n}_{0}^{2}$ (with *E* = |**E**|), these can be approximated as

2 $${n}_{1,2}(t)={n}_{0}+\frac{{n}_{0}^{3}{r}_{41}E(t)}{4}(\sin \,\alpha \pm \sqrt{1+3\,{\cos }^{2}\,\alpha }).$$

A linearly polarized laser (with wavelength *λ*_*L*_), passing through an electro-optically modulated ZnTe crystal of thickness *d*, experiences a modulation in its polarization and its electric field components (projected along the principal axes **U**_1,2_) receive a relative phase shift

3 $${\rm{\Gamma }}(t)=\frac{2\pi ({n}_{1}-{n}_{2})d}{{\lambda }_{L}}=\frac{\pi {n}_{0}^{3}d}{{\lambda }_{L}}{r}_{41}E(t)\sqrt{1+3{\cos }^{2}\alpha }$$

linearly dependent on the amplitude of the applied electric field. According to eq. 3, if the external electric field is absent the laser pulse remains unaffected by the EO crystal and stays with its linear polarization.

### Electro-optic encoding

**Figure 9:**
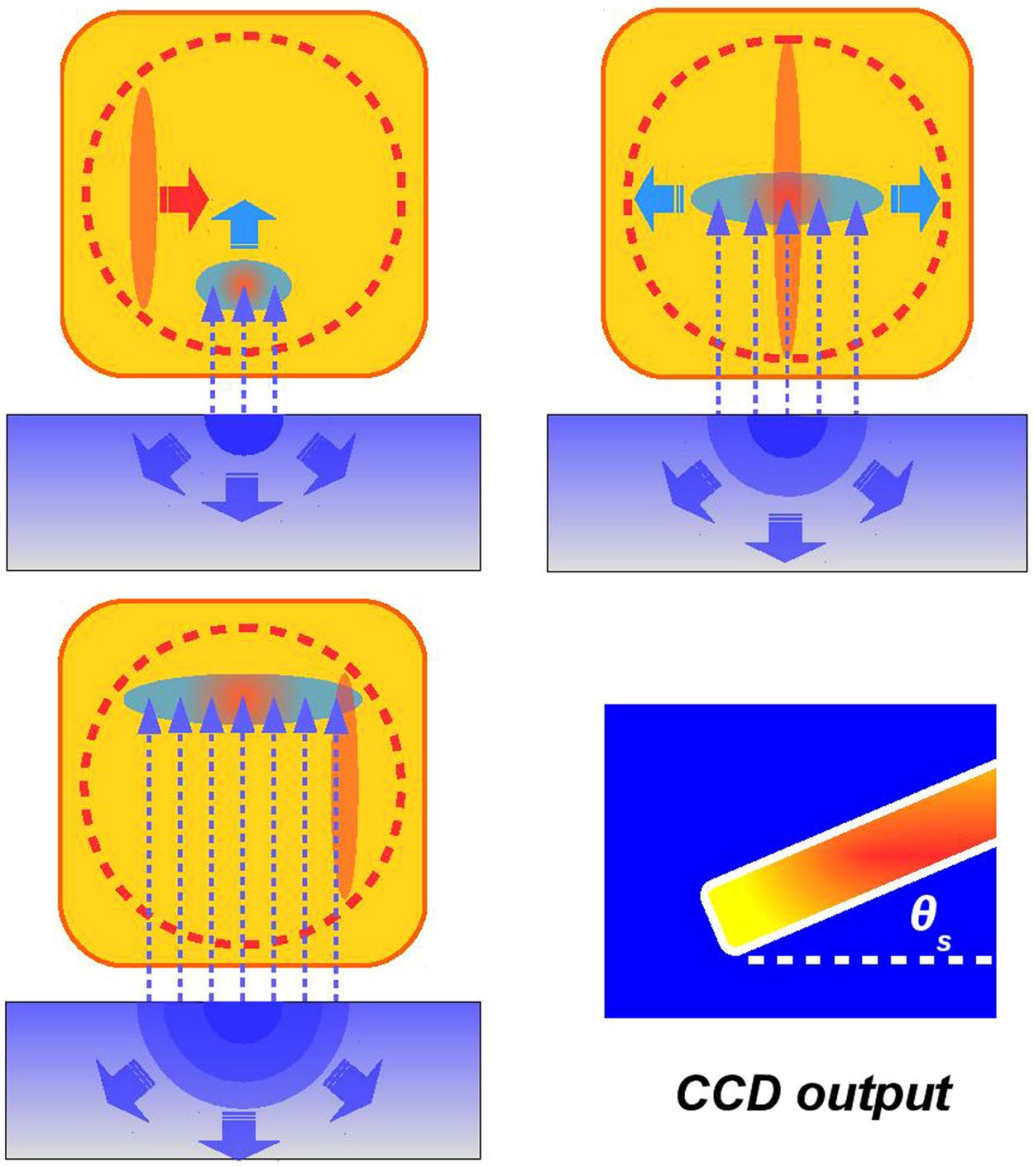
Signal detection. (**a**) The electric field (dashed arrows) impinges on the EOS crystal (yellow square) inducing electro-optic effect. (**b**) The linearly polarized probe laser (red ellipse) simultaneously crosses the crystal with an angle, spanning an overall region equal to its transverse spot size (red dashed circle). (**c**) While the charge spreads on target, the electric field source grows in size covering a larger area. (**d**) The probe polarization is locally modulated (red rectangle, rotated by *θ*_*s*_) when the probe laser temporally overlaps the radiated pulse.

There are several ways of detection of the electro-optically modulated laser. In our setup the probe laser pulse crosses the (110)-cut ZnTe crystal (500 *μm*-thickness) with *θ*_*i*_ = 28° incidence angle, realizing a spatial encoding of the radiated pulse along the laser probe transverse profile^41^. In such a way the temporal coordinate *t*_*i*_ of the pulse radiated by the target is related to the laser transverse one *x*_*i*_ as *t*_*i*_ = *x*_*i*_sin*θ*_*i*_/*c*. The electro-optic encoding process is illustrated in Fig. 9. Being 6 mm the diameter of the probe laser, the resulting active time window provided by the EOS is approximately 10 ps.

To retrieve the electric field that is actually encoded into the EOS probe laser we used a polarizer downstream the ZnTe crystal in order to convert the modulation in the polarization of the probe laser in a modulation of its intensity. The polarizer is crossed with respect to the polarization of the input laser, therefore no light transmitted in the absence of an EO-induced phase retardation. The crystal **Y** axis is put parallel to the metallic blade realizing, according to Fig. 8, *α* = 0° and Ψ = 45°. In this configuration, being *E*_*probe*_ ≈ 2 *μ*J the laser probe energy upstream the crystal, the resulting signal energy in the photo-detector (the CCD camera in our case) is given by ${E}_{det}(t)={E}_{probe}{\sin }^{2}({\rm{\Gamma }}(t)/2)$. In order to reproduce the EOS response with the current experimental setup we have developed a numerical simulation code. It assumes that, at the beginning, all the charge is concentrated within a circular region of the target approximately corresponding to the laser focal spot. The charge, with time, gradually spreads on target with its radius growing approximately the speed of light (see Fig. 4). The simulation proceeds by taking into account the dispersive propagation of the field in the ZnTe crystal^49^. Here the sampling is performed by a co-propagating probe laser pulse whose initial linear polarization gradually becomes elliptical due to the electro-optic effect that is induced, according to eq. 3, by the external electric field. The process terminates by simulating the signal output on the CCD camera. More details about the numerical simulation of the EOS response are reported in our previous work^40^.

According to eq. 3, the magnitude of the external electric field (in correspondence of the ZnTe crystal) can thus be extrapolated from the amplitude of the detected signal. This aspect allows, in turn, to calculate the corresponding field amplitude directly on target by knowing the exact distance between the target and the location of the crystal where the signal is actually detected. Since in all the measurements acquired during the experiment the electro-optic signals were emitted by the top-right part of the crystal image plane, the distance between the target and such position corresponds to $r=\sqrt{{d}^{2}+{(d+x)}^{2}}$≈ 6.2 mm (with *d* ≈ 1 mm and *x* the relative position of the signal emitting point).

### Target characterization

**Figure 10:**
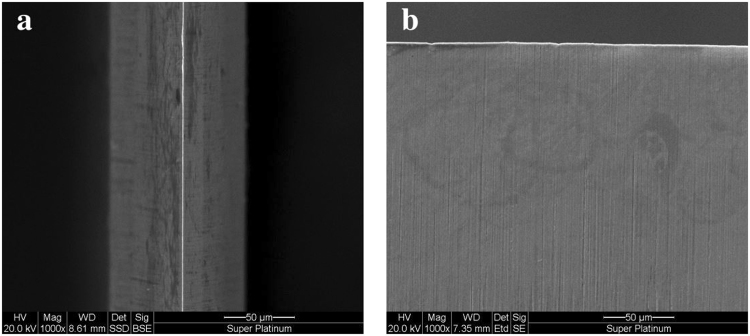
ESEM measurements of target. (**a**) Top and (**b**) front view of the razor blade used in the experiment.

The high resolution study of the razor blade structure was done by imaging it using an Environmental Scanning Electron Microscope (ESEM) Quanta 200 provided by FEI. The tip of the razor blade used in the experiment is 0.70 ± 0.05 *μm* with an opening angle of 17° ± 3°. A detailed picture of the blade used in the experiment is reported in Fig. 10. Figure 10(a) shows a top view of the blade tip while in Fig. 10(b) we report a front perspective showing its micron-scale target local perturbations.

### Particle-In-Cell Simulations

The interaction of a high-intensity short-pulse laser with metallic targets is modeled by using the 2D particle-in-cell (PIC) code TURBOWAVE^48^. The numerical simulations include a detailed description of the interaction near the surface and consist in a surface with micron-scale target local perturbations (consistent with ESEM images of the blade material used in the experiment) interacting with laser intensity of 10^18^ W cm^−2^, a spot diameter of 10 *μm* and 30 fs duration. The simulation region is 40 × 40 *μm*^2^ with 10^−2^ *μm* cell size in order to reproduce the surface roughness. Considering the pre-pulse effect on the target, the blade is considered as a Fe^5+^ plasma and each cell of the plasma region contains initially 512 particles. This resolution enables a proper description of the expected high gradients in the density and generated potential.
